# Phylogenetic Diversity of *aprA* Genes in Subseafloor Sediments on the Northwestern Pacific Margin off Japan

**DOI:** 10.1264/jsme2.ME15023

**Published:** 2015-07-04

**Authors:** Masataka Aoki, Ryota Kakiuchi, Takashi Yamaguchi, Ken Takai, Fumio Inagaki, Hiroyuki Imachi

**Affiliations:** 1 Department of Subsurface Geobiological Analysis and Research (D-SUGAR), Japan Agency for Marine-Earth Science and Technology (JAMSTEC), 2–15 Natsushima-cho, Yokosuka, Kanagawa 237–0061, Japan; 2 Department of Environmental Systems Engineering, Nagaoka University of Technology, 1603–1 Kamitomioka, Nagaoka, Niigata 940–2188, Japan; 3 Department of Construction Systems Engineering, Anan National College of Technology, 265 Aoki Minobayashi, Anan, Tokushima 774–0017, Japan; 4 Geomicrobiology Group, Kochi Institute for Core Sample Research, JAMSTEC, Monobe B200, Nankoku, Kochi 783–8502, Japan

**Keywords:** *aprA* gene, sulfate reduction, sulfur oxidation, marine sediment

## Abstract

Markedly diverse sequences of the adenosine-5′-phosphosulfate reductase alpha subunit gene (*aprA*), which encodes a key enzyme in microbial sulfate reduction and sulfur oxidation, were detected in subseafloor sediments on the northwestern Pacific off Japan. The *aprA* gene sequences were grouped into 135 operational taxonomic units (90% sequence identity), including genes related to putative sulfur-oxidizing bacteria predominantly detected in sulfate-depleted deep sediments. Our results suggest that microbial ecosystems in the subseafloor biosphere have phylogenetically diverse genetic potentials to mediate cryptic sulfur cycles in sediments, even where sulfate is rarely present.

Subseafloor microbial ecosystems play significant biogeochemical roles in the transformation of sulfur compounds; approximately 11.3 Tmols of sulfate are microbially reduced each year, accounting for the oxidation of 12%–29% of the organic carbon flux to the seafloor (5). Some sulfate-reducing bacteria play an important role in the mitigation of methane emissions from marine sediments because they may significantly interact with archaeal anaerobic methanotrophs (ANMEs) that mediate the anaerobic oxidation of methane (AOM) (13). Dissimilatory sulfate reduction by marine sediments largely depends on the availability of sulfate supplied from seawater or underlying basaltic aquifers (6). Recent biogeochemical studies detected the presence of cryptic sulfur-metabolizing activity in sulfate-depleted subseafloor sediments in which methanogenesis was the favored metabolic pathway (8, 11, 25). On the other hand, dissimilatory sulfur oxidation occurs where reduced inorganic sulfur compounds derived from abiotic processes or dissimilatory sulfate reduction is available. In methane-seep sediment, sulfur oxidizers have been identified as primary producers due to the high sulfide fluxes derived from AOM (10).

In spite of the biogeochemical significance of sulfate reduction and sulfur oxidation in subseafloor sediments, information regarding the phylogenetic diversity and distribution of functional genes relevant to the subseafloor sulfur cycle remains limited. One useful functional marker gene used to detected the microbial sulfur cycle is the adenosine-5′-phosphosulfate (APS) reductase alpha subunit (*aprA*) gene, which encodes a key enzyme for dissimilatory sulfate reduction and sulfur oxidation (19). In the present study, we investigated the diversity of the *aprA* gene in sediment samples obtained from three distinct locations on the northwestern Pacific margin off Japan in order to obtain new insights into the microbial sulfur cycle (Table 1) (1, 2, 24, 26). The depth profiles of sulfate and methane concentrations suggested the occurrence of AOM in the sampling sites (Fig. S1). Furthermore, a continuous-flow bioreactor enrichment culture capable of AOM, which had been established using the methane-seep sediment of Site 6K949 (hereafter called 6K949 enrichment), was also examined in order to identify active, culturable sulfur-metabolizing components (2).

We detected phylogenetically diverse *aprA* genes in all the samples tested by PCR and subsequent cloning procedures. The detailed procedures of total DNA extraction, PCR amplification of the *aprA* gene, clone library construction, and phylogenetic and statistical analyses are described in the Supplementary Methods. A total of 692 *aprA* gene clones were obtained, and these genes were grouped into 135 distinct operational taxonomic units (OTUs) based on 90% nucleotide sequence identity (Fig. 1 and Table S1). The rarefaction curves for all clone libraries did not plateau (Fig. S2), and the Good’s coverage values did not reach 100% in any of the clone libraries (77%–93%; Table S2). Therefore, additional *aprA* OTUs may be recovered by additional sequencing efforts with the same PCR primer set in each clone library. The higher diversity observed at shallower (*i.e.*, 3.7 m below seafloor [mbsf] of Site C9010, and 2.5 and 5.1 mbsf of Site C9001) than greater sediment depths (*i.e.*, 15.4 mbsf of Site C9010 and 48.3 mbsf of Site C9001) suggested the suppression of phylogenetic diversity at greater sediment depths of Sites C9010 and C9001 (Fig. S2 and Table S2). Based on the phylogenetic positions, each OTU was assigned to one of the 16 phylogenetic groups, including tentatively classified uncultivated groups (Fig. 1). Clusters A to G and J to L did not contain any AprA sequences from pure cultures with validated names. The *aprA*-based community structures and putative function of each phylogenetic group are shown in Fig. 2. On the basis of the closest cultured relatives and AprA phylogeny (19), 87 and 45 OTUs were assigned to putative sulfate-reducing and sulfur-oxidizing functional groups, respectively. All 45 putative sulfur-oxidizing OTUs were classified into two phylogenetically distinct sulfur-oxidizing bacteria (SOB), AprA lineages I and II (18). However, difficulties were still associated with clearly assigning the putative sulfur-oxidizing OTUs to specific taxa, even at the family level, due to the limited number of reference *aprA* genes phylogenetically close to the OTUs and low bootstrap values (Fig. 1). A previous study proposed that the *aprBA* gene of the sulfate-reducing genus *Thermodesulfovibrio* is laterally transferred to the sulfur-oxidizing family *Chlorobiaceae* (18). Therefore, the putative functions of three OTUs affiliated with clusters E and F (*i.e.*, OTUs 44, 100, and 135) remain unclear. In all samples analyzed, low-abundance OTUs (showing less than 5.0% of the clonal abundance in each library) comprised a total of 34.9%–56.0% of the composition in each clone library.

At Site C9010 offshore of the Boso Peninsula, the family *Desulfobacteraceae*-affiliated *aprA* genes comprised 60.2% of the clone library from the sediment at 3.7 mbsf (Fig. 2). The *Desulfobacteraceae*-affiliated *aprA* genes were also detected at 15.4 mbsf, which was clearly below the sulfate-depletion depth (12.6% of the total clones). In contrast, *Desulfotomaculum*-associated cluster I was identified as the major putative sulfate-reducing lineage at that depth (28.7% of all clones). In addition to cluster I, SOB AprA lineage I was frequently detected at 15.4 mbsf (49.4% of the total clones). Among the SOB AprA lineage I-associated OTUs, OTU8, which was phylogenetically closely related to the sequences of a putative autotrophic sulfur-oxidizing gammaproteobacterial lineage of Agg47 (*e.g.*, GenBank accession number ADX05650; Fig. 1), was highly dominant at 15.4 mbsf (20.7% of all clones; Table S1) (22). *Desulfobulbaceae*, clusters G and J, and SOB AprA lineage II were not abundant, but were detected at 15.4 mbsf (each group corresponding to 1.1%–4.6% of all clones).

The family *Desulfobacteraceae* comprised 57.0% of the clone library obtained from Site C9001 off the Shimokita Peninsula at 2.5 mbsf (Fig. 2). Two uncultured lineages, clusters G and L, were also relatively abundant at 2.5 mbsf (corresponding to 12.8% and 11.6% of the clone library, respectively). *Desulfobacteraceae* was also detected as the major component at a depth of 5.1 mbsf, which was just below the sulfate-depletion depth (26.4% of all clones). Clusters C, G, and L, and SOB AprA lineage I were identified as being abundant at 5.1 mbsf (corresponding to 19.5%, 12.6%, 10.3%, and 18.4% of all clones, respectively). At a greater depth of 48.3 mbsf, SOB AprA lineage I was the most abundant (43.4% of all clones). OTU1 was phylogenetically relatively close to cultured gammaproteobacterial sulfur oxidizers (*e.g.*, *Thiocapsa marina*; Fig. 1 and Table S1) and represented the predominant SOB AprA lineage I-associated OTU at 48.3 mbsf (31.6% of all clones). *Desulfotomaculum-*associated cluster I was detected as the dominant putative sulfate-reducing component at 48.3 mbsf (17.1% of all clones). In addition, other phylogenetically diverse *aprA* genes affiliated with the putative sulfate reducer lineages of *Desulfobacteraceae, Desulfobulbaceae*, and clusters G, H, and I were also detected at 48.3 mbsf (each group corresponding to 3.9%–13.2% of all clones).

*Desulfobacteraceae* and SOB AprA lineages I and II were frequently detected at Site 6K949 in the methane-seep sediment of the Nankai Trough (corresponding to 25.0%, 34.5%, and 19.8% of all clones, respectively; Fig. 2). Other putative sulfate-reducing lineages of *Desulfobulbaceae*, and clusters A, B, C, G, H, I, and L were also detected with a relatively low frequency (each group corresponded to 0.9%–6.0% of all clones). The existence of markedly diverse *aprA* genes in the 6K949 enrichment (Fig. 2 and Table S2) suggested that phylogenetically diverse microorganisms possessing the *aprA* gene at Site 6K949 were culturable under certain laboratory conditions. SOB AprA lineage I-associated OTU1 was the highly dominant OTU in the enrichment (31.8% of all clones; Table S1). The frequent detection of putative sulfur oxidizer-affiliated *aprA* genes may be linked to the input of barely oxygenated artificial seawater medium and potential sulfide production via an active AOM reaction, as described in our previous study (2). In contrast, we were unable to completely exclude potential anaerobic growth by putative sulfur oxidizers colonized in the enrichment (*e.g.*, disproportionation of inorganic sulfur compounds; see later).

The frequent detection of putative sulfate-reducing *aprA* genes in shallow sediment depths associated with the typical sulfate-reducing zone is not surprising. In the present study, *Desulfobacteraceae*-associated *aprA* genes were identified as the major components at Site 6K949 and shallow sediment depths of Sites C9010 and C9001 (Fig. 2). The dominance of *Desulfobacteraceae* has also been observed in geographically distinct subseafloor environments (4, 17). Most cultured members of the family *Desulfobacteraceae* completely oxidize a wide range of organic compounds to carbon dioxide with sulfate or other inorganic sulfur compounds as the electron acceptor (15). Growth with fermentation (15), iron reduction (15), the disproportionation of inorganic sulfur compounds (15, 20), gaseous short-chain alkane degradation using sulfate (12), and lithoautotrophic growth with hydrogen (15) have been reported in *Desulfobacteraceae* members. Therefore, *Desulfobacteraceae* sulfate reducers appear to be capable of occupying ecological niches in diverse marine environments due to their metabolic versatility. The symbiotic lifestyle of the ANME-2 group with the *Desulfosarcina*/*Desulfococcus* (DSS) group within *Desulfobacteraceae* has been reported previously (13). According to a mechanism recently proposed by Milucka *et al.*(20), DSS disproportionates zero-valent sulfur (in the form of disulfide) formed by ANME-2 to sulfate and sulfide in a sulfate-driven AOM reaction. OTU2, which was frequently detected at Sites C9010 and 6K949, was phylogenetically close to environmental sequences retrieved from an ANME-2/DSS-dominated microbial mat (Fig. 1). Thus, the presence of some *Desulfobacteraceae aprA* genes may be solely attributed to sulfate-driven AOM activity.

We also detected phylogenetically diverse putative sulfate-reducing *aprA* genes in sulfate-depleted (<1 mM sulfate) sediment horizons below the typical sulfate-reducing zone (Fig. 2). These results suggested that phylogenetically diverse putative sulfate reducers exhibited high sulfate affinity to adapt to low-sulfate subseafloor environments (23). Among the phylogenetically diverse putative sulfate-reducing groups, *Desulfotomaculum*-associated cluster I was frequently detected at greater depths. Most cultured members of the genus *Desulfotomaculum* have the ability to oxidize various organic substances with sulfate or other inorganic sulfur compounds as the terminal electron acceptor (3, 14). Growth with fermentation (3, 14), metal reduction (3, 14), thiosulfate disproportionation (3, 7), syntrophic association with methanogens in the absence of sulfate (9), propane degradation using sulfate (12), and lithoautotrophic growth with hydrogen (3, 14) have been reported in *Desulfotomaculum*-related lineages. Another unique physiological feature of *Desulfotomaculum* members is their sporulation capability (3, 14). Sporulation is considered to be one of the microbial strategies used to resist nutrient deprivation, unfavorable temperatures, or redox conditions (3). Assuming that cluster I-affiliated members share *Desulfotomaculum*-like versatile physiological features, these members may have advantages for long-term survival in deep subseafloor environments in which nutrient availability is severely limited (10). In contrast, previously identified *Desulfotomaculum* members have not yet been detected and isolated from deep subseafloor environments (3). Further analyses are needed in order to obtain detailed genetic and physiological information on putative sulfate reducers possessing the cluster I *aprA* gene.

Another interesting result of this functional gene survey was the frequent detection of SOB AprA lineage I-affiliated *aprA* genes in sulfate-depleted deep subseafloor sediment samples at Sites C9010 and C9001 (Fig. 2). Furthermore, our phylogenetic analysis suggested that some as yet uncharacterized sulfur oxidizers potentially affiliated with the class *Gammaproteobacteria* may function in the biological sulfur cycle at greater depths. Although we did not obtain quantitative data regarding general terminal electron acceptors for sulfur oxidizers (*i.e.*, oxygen, nitrate, and nitrite), these electron acceptors may be available less at sulfate-depleted depths because of higher reduction potential ranges than those of sulfate (16). In contrast, mRNA transcripts coding for dissimilatory nitrate reductases were expressed at the sulfate-depleted sediment depths of the Peru Margin sediment (Site 1229, Hole D) in the absence of measureable nitrate (21). Thus, the presence of trace amounts of nitrate at sulfate-depleted lower-redox depths may be one of the conceivable explanations for the detection of putative sulfur oxidizer-related *aprA* genes. Another possible metabolism that may sustain the putative sulfur oxidizers possessing the *aprA* gene is the disproportionation of inorganic sulfur compounds, such as thiosulfate (S_2_O_3_^2−^ + H_2_O → SO_4_^2−^ + HS^−^ + H^+^) and elemental sulfur (4S^0^ + 4H_2_O → SO_4_^2−^ + 3HS^−^ + 5H^+^) (7). Reactive metal species (*e.g.*, Mn(IV) and Fe(III)), which may be involved in the abiotic oxidation of sulfide to thiosulfate and that to elemental sulfur, have been detected at sulfate-depleted-sediment depths of diverse marine sediments (8, 11). Therefore, the disproportionation of inorganic sulfur compounds may occur as primary energy-yielding metabolism in the sulfate-depleted subseafloor sediment once reactive metal species are available; however, elemental sulfur disproportionation requires the removal of the produced sulfide to be thermodynamically favorable (7). OTU5, phylogenetically closely related to the genus *Desulfocapsa*, which contains isolates capable of the disproportionation of inorganic sulfur compounds (7), was one of the frequently detected OTUs at 48.3 mbsf of Site C9001 (10.5% of all clones; Table S1).

In conclusion, our functional gene survey suggested the presence of markedly diverse *aprA* sequences in subseafloor sediments on the northwestern Pacific margin off Japan, and also that the microbial ecosystems involved exhibited markedly diverse genetic potentials to mediate the sulfur cycle even below the typical sulfate-reducing zone. In contrast, the *in situ* expression of *aprA* genes, *in situ* activity rates, and the detailed physiological properties of microorganisms possessing the *aprA* gene currently remain unclear. The combination of an *in situ* metatranscriptomic analysis and laboratory cultivation studies will provide deeper insights into the potential biogeochemical sulfur cycle in subseafloor microbial ecosystems.

The *aprA* nucleotide sequences reported in this study have been deposited in the DDBJ/EMBL/GenBank database under accession numbers LC015806–LC016497.

## Supplementary Information



## Figures and Tables

**Fig. 1:**
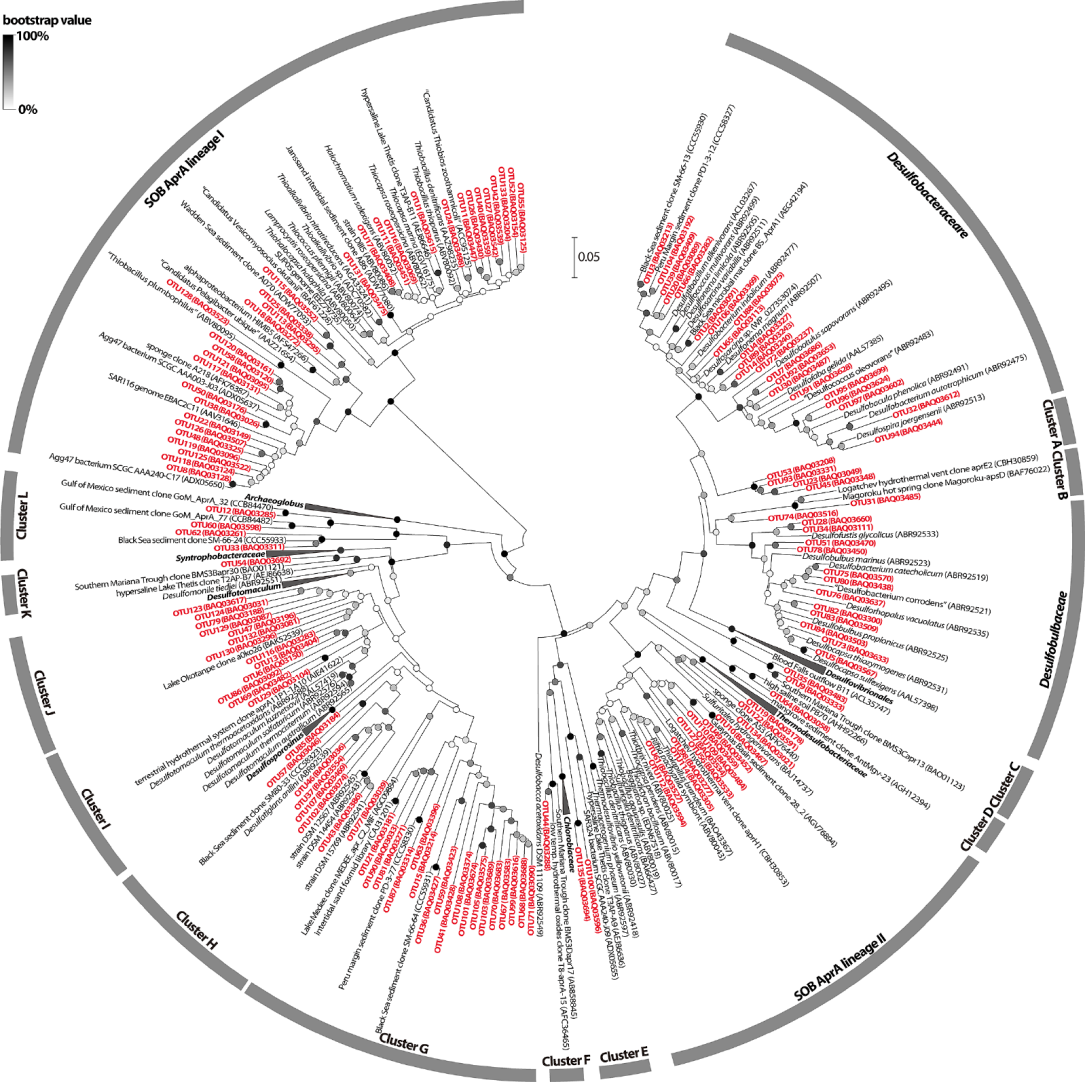
Neighbor-joining phylogenetic tree of deduced AprA amino acid sequences. Bold red letters indicate the OTUs detected in this study. The accession numbers are shown in parentheses. The scale bar represents 0.05 amino acid substitutions per sequence position. The color of circles at the branch nodes indicates the bootstrap values obtained after 1,000 resamplings.

**Fig. 2:**
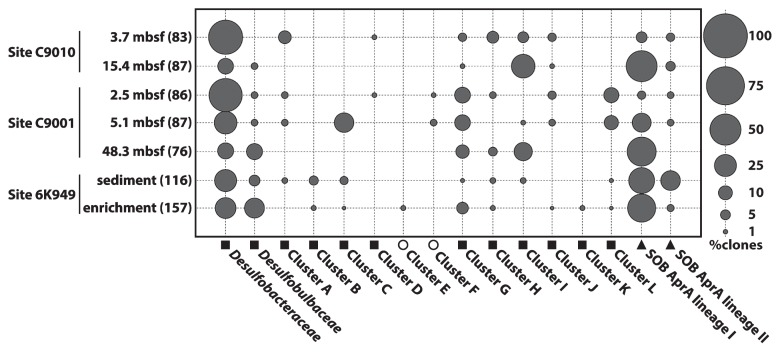
Community structures based on *aprA* gene clone libraries. The size of each dot indicates the percentage of identified *aprA* gene sequences falling within a particular taxonomic group. The symbols before the group names indicate the putative function in each taxonomic group. Closed squares, closed triangles, and open circles indicate sulfate reduction, sulfur oxidation, and uncertain function, respectively. The numbers of obtained clones in each library are shown in parentheses.

**Table 1:** Summary of sediment samples used in this study

Location	Offshore Boso Peninsula	Offshore Shimokita Peninsula	Nankai Trough
Site (hole name)	C9010 (Hole E)	C9001 (Hole C)	6K949^b^
Cruise	CK09-03	CK06-06	YK06-03
Latitude	34°33.4569′N	41°10.6380′N	33°7.2253′N
Longitude	139°53.3822′E	142°12.081′E	136°28.6672′E
Water depth [mbsl]	2,027	1,180	2,533
Sampling depth [mbsf]	3.7 and 15.4^a^	2.5, 5.1, and 48.3^a^	0–0.25^c^
Reference	(26)	(1)	(2, 24)

mbsl: m below sea level, mbsf: m below seafloor.

a These are the sampling depths of 10-cm-long whole-round cores.

b This is the sampling site of core 949C3 (2, 24).

c Subsumpled sediment analyzed in this study is same as the inoculum used for bioreactor enrichment, and the enrichment culture analyzed in this study was collected after 903 days after initiation of the bioreactor operation (2).
