# Supplementary material for: Phylogenetic Diversity of aprA Genes in Subseafloor Sediments on the Northwestern Pacific Margin off Japan
Source: Microbes Environ. 2015 Jul 4;30(3):276–80. doi: 10.1264/jsme2.ME15023 (PMC4567568; doi:10.1264/jsme2.ME15023)
Supplement: Supplementary file 1 [file 30_276_s1.pdf]

**Supplementary Information for "Phylogenetic diversity of *aprA* genes in subseafloor sediments on the northwestern Pacific margin off Japan"**

**Masataka Aoki, Ryota Kakiuchi, Takashi Yamaguchi, Ken Takai, Fumio Inagaki, and Hiroyuki Imachi**

## Supplementary Methods

**Total DNA extraction, PCR amplification, clone library construction and sequencing.** Extraction of total DNA was performed using an ISOIL for Beads Beating kit (Nippon Gene, Tokyo, Japan). All the natural sediment samples for the clone library construction were subsampled from the innermost of the sediment cores. All the samples were stored at −80°C after subsampling until total DNA extraction was performed. PCR amplification was performed using the TaKaRa Ex Taq (TaKaRa Bio Inc., Otsu, Japan), and the reaction mixtures for PCR were prepared according to the manufacturer's instruction. *aprA* gene fragments were amplified using primer pair AprA-1-FW (5'-TGG CAG ATC ATG ATY MAY GG-3') and AprA-5-RV' (5'-GCG CCA ACN GGD CCR TA-3'; a slightly modified version of AprA-5-RV) (4). PCR was performed under the following conditions: initial denaturation at 95°C for 2 min, followed by 25, 30, or 35 cycles of denaturation at 95°C for 40 s, annealing at 55°C for 30 s, and extension at 72°C for 30 s. The final extension step was conducted at 72°C for 7 min. The annealing temperature was empirically determined using genomic DNA extracts from three sulfate-reducing bacterial strains of *Thermodesulfobacterium commune* strain DSM 2178, *Desulfotomaculum thermosapovorans* strain DSM 6562, and *Desulfovibrio vulgaris* subsp. *vulgaris* strain DSM 644 obtained from Deutsche Sammlung von Mikroorganismen und Zellkulturen GmbH, Germany. To reduce possible bias caused by PCR amplification, PCR products that had obtained the minimal number of PCR cycles were used for following experiments. The PCR products were checked on 1%–2% agarose gel electrophoresis, and the expected-size PCR products (approximately 0.4 kbp) were purified using a MinElute Gel

Extraction kit (Qiagen, Venlo, the Netherlands). After purification, the purified PCR products were cloned with a pCR 2.1 TOPO TA cloning kit (Invitrogen, Carlsbad, CA, USA). The cloned *aprA* gene sequences were determined using a BigDye terminator v3.1 cycle sequencing kit (Applied Biosystems, Foster City, CA, USA) and an automated sequence analyzer (3730xl DNA Analyzer, Applied Biosystems). Clonal sequences with stop codons were excluded from this study.

**Phylogenetic and statistical analyses.** The obtained *aprA* gene sequences were aligned using the ClustalX program (2), version 2.1, and a sequence distance matrix was generated using the ARB program (3), version 6.0. The calculated distance matrix was inputted into the mothur program (6), version 1.33 and the obtained *aprA* gene sequences were grouped into operational taxonomic units (OTUs) at a 90% nucleotide sequence identity based on the matrix. Putative chimeric sequences were not detected using the “chimera.uchime” command in the mothur program and the more abundant sequences in the obtained clone libraries as a reference. Translated deduced AprA amino acid sequences were subjected to BLASTP analysis (http://blast.ncbi.nlm.nih.gov/Blast.cgi). The deduced AprA amino acid sequences (111–127 amino acid residues) used for AprA tree construction were aligned using the ClustalX program, and then manually corrected. A neighbor-joining AprA phylogenetic tree construction was performed using the MEGA program (7), version 6.06. A pairwise distance matrix used for the tree construction was calculated based on the aligned AprA sequences and the Poisson model. A bootstrap analysis with 1,000 replicates was performed to assign confidence levels to the tree topology. The resulting tree was displayed using the FigTree program, version 1.4.2 (http://tree.bio.ed.ac.uk/software/figtree/). Chao1 and ACE richness estimators, Shannon diversity indices, Good’s coverage values, and rarefaction curves were calculated using the mothur program

**Supplementary Figure Legends**

**Fig. S1.** Vertical profiles of sulfate and methane concentrations in sediment core samples. (**a**) Site C9010, Hole E, offshore the Boso Peninsula (only shown above 35 mbsf) (10). (**b**) Site C9001, Hole C, offshore the Shimokita Peninsula (only shown above 60 mbsf) (1, 9). (**c**) Site 6K949 in the Nankai Trough (5, 8). At Site C9010, methane concentrations were only measured in the deeper layer of Hole E (below about 100 mbsf), whereas an increasing trend in methane concentrations along with the increasing depth was observed in 1–15 mbsf of Hole B (34°33.4500'N, 139°53.4000'E; about 30 m away from Hole E) (10).

**Fig. S2.** Rarefaction curves for each *aprA* gene clone library.

# Supplementary Figures

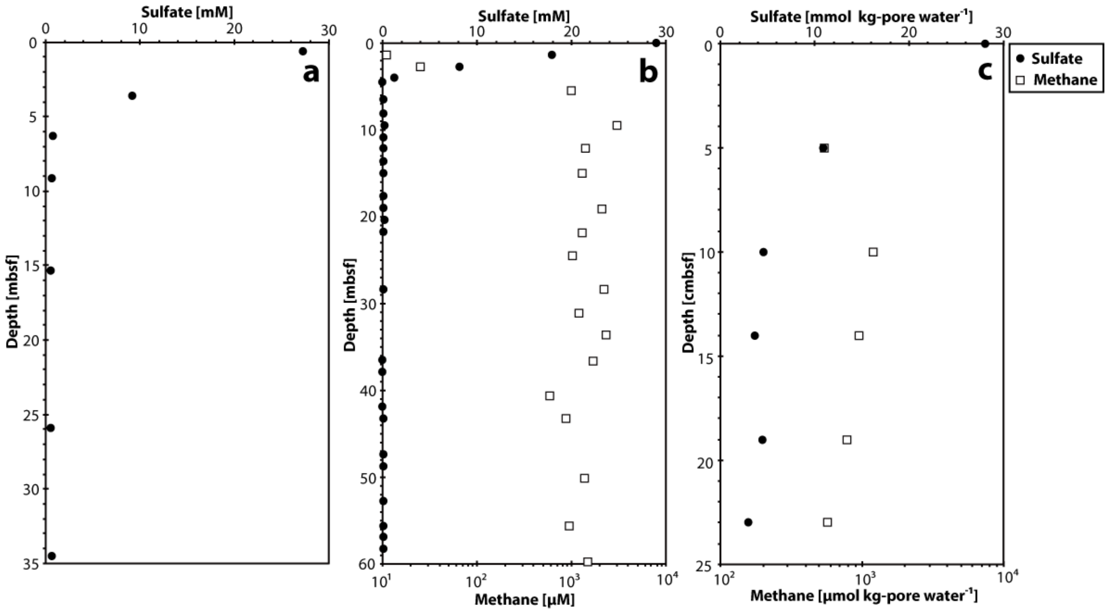


**Fig. S1. Aoki et al.**

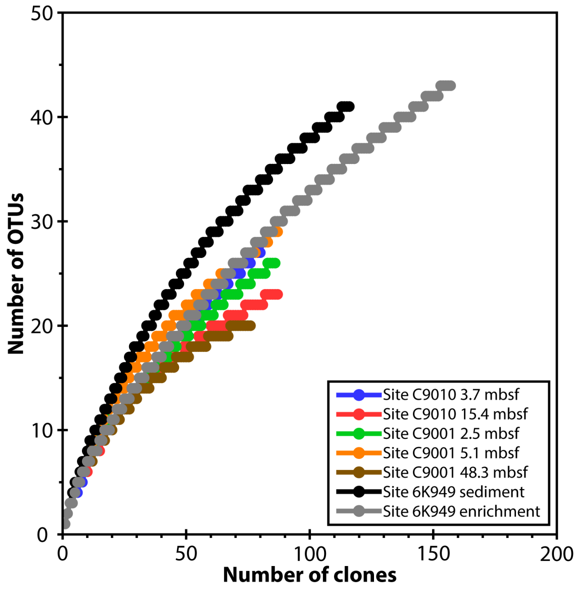


**Fig. S2. Aoki et al.**

# Supplementary Tables

**Table S1.** List of *aprA* gene OTUs detected in this study

| OTU name | Number of clones | | | | | | | total | Phylogenetic affiliation | Similar AprA sequence with validated name (GenBank accession number) | Maximum Identity [%] |
|---|---|---|---|---|---|---|---|---|---|---|---|
| | Site C9010 | | Site C9001 | | | Site 6K949 | | | | | |
| | 3.7 mbsf | 15.4 mbsf | 2.5 mbsf | 5.1 mbsf | 48.3 mbsf | sediment | enrichment | | | | |
| OTU1 | 0 | 0 | 0 | 3 | 24 | 0 | 50 | 77 | AprA lineage I | *Thiocapsa marina* (EGV16175) | 95.7 |
| OTU2 | 35 | 0 | 0 | 0 | 3 | 21 | 1 | 60 | *Desulfobacteraceae* | *Desulfosarcina variabilis* (ABR92511) | 95.8 |
| OTU3 | 5 | 1 | 22 | 4 | 2 | 2 | 6 | 42 | *Desulfobacteraceae* | *Desulfatibacillum alkenivorans* (ACL03267) | 93.3 |
| OTU4 | 3 | 6 | 12 | 13 | 0 | 0 | 0 | 34 | *Desulfobacteraceae* | *Desulfonema magnum* (ABR92507) | 96.6 |
| OTU5 | 0 | 0 | 0 | 1 | 8 | 1 | 22 | 32 | *Desulfobulbaceae* | *Desulfocapsa sulfexigens* (AAL57398) | 97.5 |
| OTU6 | 1 | 24 | 0 | 0 | 2 | 0 | 0 | 27 | Cluster I | *Desulfotomaculum australicum* (ABR92565) | 89.9 |
| OTU7 | 0 | 0 | 0 | 4 | 2 | 0 | 17 | 23 | *Desulfobacteraceae* | *Desulfofaba gelida* (AAL57385) | 95.8 |
| OTU8 | 1 | 18 | 0 | 0 | 0 | 0 | 0 | 19 | AprA lineage I | *Thioalkalivibrio sulfidiphilus* (ACL71448) | 80.3 |
| OTU9 | 0 | 0 | 0 | 16 | 0 | 2 | 0 | 18 | Cluster C | *Sedimenticola selenatireducens* (WP_029134892) | 79.8 |
| OTU10 | 0 | 0 | 0 | 0 | 0 | 13 | 2 | 15 | AprA lineage II | *Thiobacillus thioparus* (ABV80027) | 91.6 |
| OTU11 | 0 | 0 | 0 | 0 | 1 | 11 | 1 | 13 | AprA lineage I | *Thiobacillus denitrificans* (WP_011312794) | 96.6 |
| OTU12 | 0 | 0 | 8 | 5 | 0 | 0 | 0 | 13 | Cluster L | *Desulfotomaculum gibsoniae* (ABR92567) | 70.6 |
| OTU13 | 0 | 0 | 0 | 0 | 11 | 0 | 0 | 11 | Cluster I | *Desulfotomaculum australicum* (ABR92565) | 92.4 |
| OTU14 | 0 | 0 | 9 | 0 | 0 | 0 | 2 | 11 | *Desulfobacteraceae* | *Desulfosarcina variabilis* (AAL57388) | 96.6 |
| OTU15 | 0 | 0 | 5 | 5 | 0 | 0 | 0 | 10 | Cluster G | *Desulfotomaculum australicum* (ABR92565) | 76.7 |
| OTU16 | 1 | 0 | 1 | 0 | 4 | 3 | 1 | 10 | AprA lineage I | *Thiocapsa roseopersicina* (ABV80060) | 94.9 |
| OTU17 | 0 | 0 | 0 | 0 | 2 | 0 | 7 | 9 | AprA lineage I | *Halochromatium salexigens* (ABV80052) | 96.6 |
| OTU18 | 0 | 4 | 0 | 5 | 0 | 0 | 0 | 9 | AprA lineage I | *Thiobacillus denitrificans* (WP_011312794) | 82.1 |
| OTU19 | 2 | 4 | 2 | 0 | 0 | 0 | 0 | 8 | AprA lineage II | *Sulfuritalea hydrogenivorans* sk43H (BAJ14737) | 84.0 |
| OTU20 | 5 | 2 | 0 | 1 | 0 | 0 | 0 | 8 | *Desulfobacteraceae* | *Desulfonema ishimotonii* (AAL57402) | 93.3 |
| OTU21 | 0 | 0 | 4 | 3 | 0 | 0 | 0 | 7 | Cluster G | *Desulfotomaculum australicum* (ABR92565) | 79.8 |
| OTU22 | 1 | 5 | 0 | 0 | 0 | 0 | 0 | 6 | AprA lineage I | *Thioalkalivibrio sulfidiphilus* (ACL71448) | 80.3 |
| OTU23 | 6 | 0 | 0 | 0 | 0 | 0 | 0 | 6 | Cluster A | *Desulfonema magnum* (ABR92507) | 88.2 |
| OTU24 | 0 | 0 | 0 | 0 | 0 | 6 | 0 | 6 | AprA lineage I | *Thioalkalivibrio thiocyanodenitrificans* (WP_018233553) | 96.6 |
| OTU25 | 0 | 0 | 0 | 4 | 0 | 2 | 0 | 6 | AprA lineage I | *Chromatium okenii* (ABV80056) | 83.8 |
| OTU26 | 0 | 0 | 0 | 0 | 0 | 4 | 2 | 6 | AprA lineage I | *Halochromatium salexigens* (ABV80052) | 94.9 |
| OTU27 | 0 | 0 | 0 | 0 | 0 | 5 | 1 | 6 | AprA lineage I | *Thiocapsa roseopersicina* (ABV80060) | 96.6 |
| OTU28 | 0 | 0 | 0 | 0 | 2 | 0 | 3 | 5 | *Desulfobulbaceae* | *Desulforhopalus singaporensis* (AAL57430) | 95.0 |
| OTU29 | 3 | 1 | 0 | 1 | 0 | 0 | 0 | 5 | Cluster I | *Desulfotomaculum australicum* (ABR92565) | 87.4 |
| OTU30 | 0 | 0 | 0 | 0 | 0 | 5 | 0 | 5 | *Desulfobacteraceae* | *Desulfofaba fastidiosa* (AAU95383) | 94.1 |
| OTU31 | 0 | 0 | 0 | 0 | 0 | 3 | 2 | 5 | Cluster B | *Desulfobulbus japonicus* (WP_028580570) | 82.6 |
| OTU32 | 0 | 0 | 0 | 0 | 0 | 0 | 4 | 4 | *Desulfobacteraceae* | *Desulfobacterium autotrophicum* (ABR92475) | 94.1 |
| OTU33 | 0 | 0 | 0 | 4 | 0 | 0 | 0 | 4 | Cluster L | *Desulfovirgula thermocuniculi* (WP_027718700) | 69.4 |
| OTU34 | 0 | 2 | 2 | 0 | 0 | 0 | 0 | 4 | *Desulfobulbaceae* | *Desulforhopalus singaporensis* (AAL57430) | 96.6 |
| OTU35 | 0 | 0 | 0 | 1 | 0 | 2 | 1 | 4 | Cluster C | *Desulfurivibrio alkaliphilus* (ADH86166) | 79.0 |
| OTU36 | 0 | 1 | 1 | 0 | 1 | 0 | 1 | 4 | Cluster G | *Desulfotomaculum australicum* (ABR92565) | 75.8 |
| OTU37 | 0 | 0 | 1 | 0 | 0 | 1 | 2 | 4 | Cluster H | *Desulfatiglans anilini* (AAL57425) | 86.3 |
| OTU38 | 1 | 3 | 0 | 0 | 0 | 0 | 0 | 4 | AprA lineage I | *Thioalkalivibrio sulfidiphilus* (ACL71448) | 81.2 |
| OTU39 | 1 | 0 | 1 | 0 | 2 | 0 | 0 | 4 | *Desulfobacteraceae* | *Desulfobacterium indolicum* (ABR92477) | 94.1 |
| OTU40 | 0 | 0 | 0 | 0 | 2 | 1 | 1 | 4 | AprA lineage I | *Halochromatium salexigens* (ABV80052) | 94.0 |
| OTU41 | 0 | 0 | 1 | 0 | 2 | 0 | 0 | 3 | Cluster G | *Desulfotomaculum australicum* (ABR92565) | 74.2 |
| OTU42 | 0 | 0 | 0 | 0 | 0 | 3 | 0 | 3 | AprA lineage I | *Halochromatium salexigens* (ABV80052) | 94.0 |
| OTU43 | 0 | 0 | 0 | 0 | 3 | 0 | 0 | 3 | Cluster H | *Desulfatiglans anilini* (AAL57425) | 87.3 |
| OTU44 | 0 | 0 | 1 | 2 | 0 | 0 | 0 | 3 | Cluster F | *Chlorobium clathratiforme* (ABV79999) | 78.7 |

**Table S1**. —continued

| | | | | | | | | | | | |
|---|---|---|---|---|---|---|---|---|---|---|---|
| OTU45 | 1 | 0 | 0 | 1 | 0 | 1 | 0 | 3 | Cluster A | *Desulfonema ishimotonii* (AAL57402) | 87.4 |
| OTU46 | 3 | 0 | 0 | 0 | 0 | 0 | 0 | 3 | Cluster H | *Desulfatiglans anilini* (AAL57425) | 85.5 |
| OTU47 | 0 | 1 | 2 | 0 | 0 | 0 | 0 | 3 | Cluster J | *Desulfotomaculum australicum* (ABR92565) | 84.0 |
| OTU48 | 0 | 0 | 0 | 3 | 0 | 0 | 0 | 3 | AprA lineage I | *Thiorhodovibrio winogradskyi* (ABV80076) | 80.3 |
| OTU49 | 0 | 0 | 0 | 0 | 0 | 3 | 0 | 3 | AprA lineage II | *Thiobacillus thioparus* (ABV80027) | 89.1 |
| OTU50 | 0 | 3 | 0 | 0 | 0 | 0 | 0 | 3 | AprA lineage I | *Thioalkalivibrio sulfidophilus* (ACL71448) | 79.5 |
| OTU51 | 0 | 0 | 0 | 0 | 0 | 2 | 1 | 3 | *Desulfobulbaceae* | *Desulfobulbus marinus* (ABR92523) | 91.6 |
| OTU52 | 1 | 2 | 0 | 0 | 0 | 0 | 0 | 3 | AprA lineage I | *Halochromatium salexigens* (ABV80052) | 94.0 |
| OTU53 | 0 | 0 | 2 | 0 | 0 | 1 | 0 | 3 | Cluster A | *Desulfonema ishimotonii* (AAL57402) | 90.8 |
| OTU54 | 0 | 0 | 0 | 0 | 0 | 0 | 2 | 2 | Cluster K | *Desulfotomaculum intricatum* (BAN14411) | 81.0 |
| OTU55 | 0 | 1 | 1 | 0 | 0 | 0 | 0 | 2 | AprA lineage I | *Thiobacillus denitrificans* (WP_011312794) | 94.9 |
| OTU56 | 0 | 0 | 0 | 0 | 0 | 2 | 0 | 2 | AprA lineage II | *Thiobacillus thioparus* (ABV80027) | 84.9 |
| OTU57 | 2 | 0 | 0 | 0 | 0 | 0 | 0 | 2 | Cluster H | *Desulfotomaculum thermobenzoicum* (AAL57428) | 81.8 |
| OTU58 | 0 | 2 | 0 | 0 | 0 | 0 | 0 | 2 | AprA lineage I | *Thioalkalivibrio sulfidophilus* (YP_002512435) | 80.3 |
| OTU59 | 0 | 0 | 0 | 1 | 1 | 0 | 0 | 2 | Cluster G | *Desulfotomaculum australicum* (ABR92565) | 80.0 |
| OTU60 | 0 | 0 | 0 | 0 | 0 | 1 | 1 | 2 | Cluster L | *Desulfotomaculum gibsoniae* (ABR92567) | 68.6 |
| OTU61 | 0 | 0 | 0 | 0 | 0 | 2 | 0 | 2 | AprA lineage II | *Thiolapillus brandeum* (BAO43367) | 95.0 |
| OTU62 | 0 | 0 | 2 | 0 | 0 | 0 | 0 | 2 | Cluster L | *Desulfomonile tiedjei* (AAL57429) | 65.3 |
| OTU63 | 0 | 0 | 0 | 0 | 2 | 0 | 0 | 2 | Cluster G | *Desulfotomaculum australicum* (ABR92565) | 80.0 |
| OTU64 | 1 | 0 | 1 | 0 | 0 | 0 | 0 | 2 | Cluster D | *Desulfonatronovibrio hydrogenovorans* (ABR92460) | 74.0 |
| OTU65 | 0 | 2 | 0 | 0 | 0 | 0 | 0 | 2 | *Desulfobacteraceae* | *Desulfobacterium indolicum* (ABR92477) | 95.0 |
| OTU66 | 0 | 0 | 1 | 1 | 0 | 0 | 0 | 2 | *Desulfobacteraceae* | *Desulfococcus multivorans* (AAL57403) | 92.4 |
| OTU67 | 0 | 0 | 0 | 0 | 0 | 0 | 2 | 2 | Cluster G | *Desulfotomaculum australicum* (ABR92565) | 80.0 |
| OTU68 | 0 | 0 | 0 | 0 | 0 | 0 | 2 | 2 | Cluster G | *Desulfotomaculum australicum* (ABR92565) | 81.7 |
| OTU69 | 0 | 0 | 0 | 0 | 0 | 2 | 0 | 2 | Cluster I | *Desulfotomaculum australicum* (ABR92565) | 86.6 |
| OTU70 | 0 | 0 | 0 | 0 | 0 | 0 | 2 | 2 | Cluster G | *Desulfotomaculum australicum* (ABR92565) | 80.0 |
| OTU71 | 1 | 0 | 0 | 0 | 0 | 1 | 0 | 2 | Cluster G | *Desulfotomaculum australicum* (ABR92565) | 80.0 |
| OTU72 | 0 | 0 | 2 | 0 | 0 | 0 | 0 | 2 | *Desulfobacteraceae* | *Desulfosarcina variabilis* (AAL57388) | 94.1 |
| OTU73 | 0 | 0 | 0 | 0 | 0 | 0 | 2 | 2 | *Desulfobulbaceae* | *Desulfocapsa thiozymogenes* (ABR92531) | 93.3 |
| OTU74 | 0 | 0 | 0 | 0 | 0 | 2 | 0 | 2 | Cluster B | *Desulfonema ishimotonii* (AAL57402) | 86.6 |
| OTU75 | 0 | 0 | 0 | 0 | 0 | 0 | 2 | 2 | *Desulfobulbaceae* | *Desulfobacterium catecholicum* (ABR92517) | 99.2 |
| OTU76 | 0 | 0 | 0 | 0 | 0 | 0 | 2 | 2 | *Desulfobulbaceae* | *Desulfobacterium catecholicum* (ABR92517) | 94.1 |
| OTU77 | 1 | 0 | 0 | 0 | 0 | 0 | 0 | 1 | Cluster G | *Ammonifex degensii* (ACX52207) | 84.2 |
| OTU78 | 0 | 0 | 0 | 0 | 0 | 1 | 0 | 1 | *Desulfobulbaceae* | *Desulfobulbus marinus* (ABR92523) | 90.8 |
| OTU79 | 0 | 0 | 1 | 0 | 0 | 0 | 0 | 1 | Cluster J | *Desulfotomaculum gibsoniae* (ABR92567) | 82.9 |
| OTU80 | 0 | 0 | 0 | 0 | 0 | 1 | 0 | 1 | *Desulfobulbaceae* | *Desulfobacterium catecholicum* (ABR92517) | 95.8 |
| OTU81 | 0 | 0 | 0 | 1 | 0 | 0 | 0 | 1 | Cluster G | *Desulfomonile tiedjei* (AAL57429) | 79.8 |
| OTU82 | 0 | 0 | 0 | 1 | 0 | 0 | 0 | 1 | *Desulfobulbaceae* | *Desulfocapsa thiozymogenes* (ABR92531) | 95.0 |
| OTU83 | 0 | 0 | 0 | 0 | 0 | 1 | 0 | 1 | *Desulfobulbaceae* | *Desulfurivibrio alkaliphilus* (ADH86166) | 94.1 |
| OTU84 | 0 | 0 | 0 | 0 | 0 | 1 | 0 | 1 | *Desulfobulbaceae* | *Desulfocapsa thiozymogenes* (ABR92531) | 94.1 |
| OTU85 | 0 | 0 | 1 | 0 | 0 | 0 | 0 | 1 | Cluster H | *Desulforhabdus amnigena* (ABR92553) | 85.7 |
| OTU86 | 1 | 0 | 0 | 0 | 0 | 0 | 0 | 1 | Cluster I | *Desulfotomaculum australicum* (ABR92565) | 84.9 |
| OTU87 | 0 | 0 | 0 | 1 | 0 | 0 | 0 | 1 | Cluster G | *Desulfomonile tiedjei* (AAL57429) | 82.2 |
| OTU88 | 1 | 0 | 0 | 0 | 0 | 0 | 0 | 1 | *Desulfobacteraceae* | *Desulfobacterium indolicum* (ABR92477) | 91.6 |
| OTU89 | 0 | 0 | 1 | 0 | 0 | 0 | 0 | 1 | *Desulfobacteraceae* | *Desulfobacterium indolicum* (ABR92477) | 92.4 |
| OTU90 | 1 | 0 | 0 | 0 | 0 | 0 | 0 | 1 | Cluster G | *Desulfomonile tiedjei* (AAL57429) | 76.3 |
| OTU91 | 0 | 0 | 0 | 0 | 0 | 0 | 1 | 1 | *Desulfobacteraceae* | *Desulfofaba fastidiosa* (AAU95383) | 92.4 |

**Table S1**. —continued

| | | | | | | | | | | | |
|---|---|---|---|---|---|---|---|---|---|---|---|
| OTU92 | 0 | 0 | 0 | 0 | 0 | 0 | 1 | 1 | *Desulfobacteraceae* | *Desulfofaba gelida* (AAL57385) | 91.6 |
| OTU93 | 0 | 0 | 0 | 1 | 0 | 0 | 0 | 1 | Cluster A | *Desulfonema ishimotonii* (AAL57402) | 89.1 |
| OTU94 | 0 | 0 | 0 | 0 | 0 | 1 | 0 | 1 | *Desulfobacteraceae* | *Desulfobacterium vacuolatum* (AAL57391) | 96.6 |
| OTU95 | 0 | 0 | 0 | 0 | 0 | 0 | 1 | 1 | *Desulfobacteraceae* | *Desulfobacula toluolica* (ABR92493) | 93.3 |
| OTU96 | 0 | 0 | 0 | 0 | 0 | 0 | 1 | 1 | *Desulfobacteraceae* | *Desulfobacula toluolica* (ABR92493) | 92.4 |
| OTU97 | 0 | 0 | 0 | 0 | 0 | 0 | 1 | 1 | *Desulfobacteraceae* | *Desulfobacula toluolica* (ABR92493) | 92.4 |
| OTU98 | 0 | 0 | 0 | 1 | 0 | 0 | 0 | 1 | AprA lineage II | *Thiothrix nivea* (EIJ35356) | 88.2 |
| OTU99 | 0 | 0 | 0 | 0 | 0 | 0 | 1 | 1 | Cluster G | *Desulfotomaculum australicum* (ABR92565) | 81.7 |
| OTU100 | 0 | 0 | 0 | 0 | 0 | 0 | 1 | 1 | Cluster E | *Chlorobaculum thiosulfatiphilum* (ABV79991) | 70.9 |
| OTU101 | 0 | 0 | 0 | 0 | 0 | 0 | 1 | 1 | Cluster G | *Desulfotomaculum thermoacetoxidans* (ABR92588) | 80.8 |
| OTU102 | 1 | 0 | 0 | 0 | 0 | 0 | 0 | 1 | Cluster H | *Desulfatiglans anilini* (AAL57425) | 82.2 |
| OTU103 | 0 | 0 | 0 | 0 | 0 | 0 | 1 | 1 | Cluster G | *Desulfotomaculum australicum* (ABR92565) | 78.3 |
| OTU104 | 0 | 0 | 1 | 0 | 0 | 0 | 0 | 1 | *Desulfobacteraceae* | *Desulfatibacillum alkenivorans* (ACL03267) | 92.4 |
| OTU105 | 0 | 0 | 0 | 0 | 0 | 0 | 1 | 1 | Cluster G | *Desulfotomaculum australicum* (ABR92565) | 80.8 |
| OTU106 | 0 | 0 | 0 | 0 | 1 | 0 | 0 | 1 | *Desulfobacteraceae* | *Desulfonema ishimotonii* (AAL57402) | 92.4 |
| OTU107 | 0 | 0 | 0 | 0 | 0 | 1 | 0 | 1 | Cluster H | *Desulfatiglans anilini* (AAL57425) | 88.1 |
| OTU108 | 0 | 0 | 0 | 0 | 1 | 0 | 0 | 1 | Cluster G | *Desulfotomaculum australicum* (ABR92565) | 79.2 |
| OTU109 | 0 | 0 | 0 | 0 | 0 | 1 | 0 | 1 | AprA lineage II | *Thiolapillus brandeum* (BAO43367) | 90.8 |
| OTU110 | 0 | 0 | 0 | 0 | 0 | 1 | 0 | 1 | AprA lineage II | *Thiobacillus denitrificans* (ABV80031) | 92.4 |
| OTU111 | 0 | 0 | 0 | 0 | 0 | 1 | 0 | 1 | AprA lineage I | *Halochromatium salexigens* (ABV80052) | 95.7 |
| OTU112 | 0 | 0 | 0 | 0 | 0 | 0 | 1 | 1 | AprA lineage II | *Sedimenticola selenatireducens* (WP_029134892) | 94.1 |
| OTU113 | 0 | 0 | 0 | 1 | 0 | 0 | 0 | 1 | AprA lineage I | *Thioalkalivibrio sulfidiphilus* (ACL71448) | 83.8 |
| OTU114 | 0 | 0 | 0 | 1 | 0 | 0 | 0 | 1 | AprA lineage II | *Thiolapillus brandeum* (BAO43367) | 95.0 |
| OTU115 | 0 | 0 | 0 | 0 | 0 | 0 | 1 | 1 | AprA lineage I | *Rhabdochromatium marinum* (ABV80078) | 82.9 |
| OTU116 | 0 | 0 | 0 | 1 | 0 | 0 | 0 | 1 | Cluster J | *Desulfotomaculum australicum* (ABR92565) | 85.7 |
| OTU117 | 0 | 1 | 0 | 0 | 0 | 0 | 0 | 1 | AprA lineage I | *Thioalkalivibrio sulfidiphilus* (ACL71448) | 78.6 |
| OTU118 | 0 | 1 | 0 | 0 | 0 | 0 | 0 | 1 | AprA lineage I | *Thiohalocapsa halophila* (ABV80050) | 78.6 |
| OTU119 | 0 | 1 | 0 | 0 | 0 | 0 | 0 | 1 | AprA lineage I | *Thioalkalivibrio nitratireducens* (AGA35241) | 79.5 |
| OTU120 | 0 | 1 | 0 | 0 | 0 | 0 | 0 | 1 | AprA lineage I | *Thioalkalivibrio sulfidophilus* (WP_012636937) | 80.3 |
| OTU121 | 0 | 1 | 0 | 0 | 0 | 0 | 0 | 1 | AprA lineage I | *Thioalkalivibrio sulfidophilus* (WP_012636937) | 80.3 |
| OTU122 | 0 | 0 | 0 | 0 | 0 | 0 | 1 | 1 | AprA lineage II | *Thioploca ingrica* (BAP57403) | 85.7 |
| OTU123 | 0 | 0 | 0 | 0 | 0 | 0 | 1 | 1 | Cluster J | *Desulfotomaculum australicum* (ABR92565) | 84.0 |
| OTU124 | 1 | 0 | 0 | 0 | 0 | 0 | 0 | 1 | Cluster J | *Desulfotomaculum gibsoniae* (ABR92567) | 84.5 |
| OTU125 | 0 | 0 | 0 | 0 | 0 | 1 | 0 | 1 | AprA lineage I | *Thioalkalivibrio sulfidophilus* (WP_012636937) | 82.9 |
| OTU126 | 0 | 0 | 0 | 0 | 0 | 1 | 0 | 1 | AprA lineage I | *Rhabdochromatium marinum* (ABV80078) | 79.5 |
| OTU127 | 0 | 0 | 0 | 0 | 0 | 1 | 0 | 1 | AprA lineage II | *Thiolapillus brandeum* (BAO43367) | 95.0 |
| OTU128 | 0 | 0 | 0 | 0 | 0 | 1 | 0 | 1 | AprA lineage I | *Thiococcus pfennigii* (ABV80074) | 76.9 |
| OTU129 | 1 | 0 | 0 | 0 | 0 | 0 | 0 | 1 | Cluster J | *Desulfotomaculum australicum* (ABR92565) | 82.2 |
| OTU130 | 0 | 0 | 0 | 1 | 0 | 0 | 0 | 1 | Cluster J | *Desulfotomaculum solfataricum* (ABR92576) | 81.5 |
| OTU131 | 0 | 0 | 0 | 0 | 0 | 1 | 0 | 1 | AprA lineage I | *Thioalkalivibrio sulfidiphilus* (ACL71448) | 95.7 |
| OTU132 | 1 | 0 | 0 | 0 | 0 | 0 | 0 | 1 | Cluster J | *Desulfotomaculum australicum* (ABR92565) | 83.2 |
| OTU133 | 0 | 0 | 1 | 0 | 0 | 0 | 0 | 1 | AprA lineage I | *Thiobacillus denitrificans* (WP_011312794) | 92.3 |
| OTU134 | 1 | 0 | 0 | 0 | 0 | 0 | 0 | 1 | AprA lineage II | *Sulfurisoma sediminicola* (BAO37334) | 83.2 |
| OTU135 | 0 | 0 | 0 | 0 | 0 | 0 | 1 | 1 | Cluster E | *Chlorobium clathratiforme* (ABV79999) | 73.2 |

**Table S2.** Diversity statistics based on *aprA* gene clone libraries

| Sample | Site C9010 | | Site C9001 | | | Site 6K949 | |
|---|---|---|---|---|---|---|---|
| | 3.7 mbsf | 15.4 mbsf | 2.5 mbsf | 5.1 mbsf | 48.3 mbsf | sediment | enrichment |
| Total clone number | 83 | 87 | 86 | 87 | 76 | 116 | 157 |
| OTUs | 28 | 23 | 26 | 29 | 20 | 41 | 43 |
| Chao1 richness estimator[a] | 85 (45–219) | 31 (25–55) | 39 (30–72) | 89 (46–245) | 21 (20–28) | 62 (48–101) | 64 (51–100) |
| ACE richness estimator[a] | 105 (69–173) | 33 (26–58) | 83 (57–131) | 49 (36–89) | 24 (21–38) | 67 (51–107) | 85 (60–145) |
| Shannon diversity index[a] | 2.44 (2.12–2.77) | 2.49 (2.24–2.74) | 2.64 (2.39–2.89) | 2.91 (2.69–3.13) | 2.44 (2.18–2.70) | 3.19 (2.98–3.40) | 2.75 (2.51–2.99) |
| Good's coverage value (%) | 77 | 89 | 84 | 82 | 93 | 82 | 85 |

[a]Numbers in parentheses indicate the 95% confidence interval.

**References for Supplementary Information**

1. Aoike, K. 2007. CK06-06 D/V *Chikyu* shakedown cruise offshore Shimokita laboratory operation report. CDEX-JAMSTEC, Yokohama.

2. Larkin, M.A., G. Blackshields, N.P. Brown, et al. 2007. Clustal W and Clustal X version 2.0. Bioinformatics 23:2947-2948.

3. Ludwig, W., O. Strunk, R. Westram, et al. 2004. ARB: a software environment for sequence data. Nucleic Acids Res. 32:1363–1371.

4. Meyer, B., and J. Kuever. 2007. Molecular analysis of the diversity of sulfate-reducing and sulfur-oxidizing prokaryotes in the environment, using *aprA* as functional marker gene. Appl. Environ. Microbiol. 73:7664-7679.

5. Nunoura, T., Y. Takaki, H. Kazama, M. Hirai, J. Ashi, H. Imachi, and K. Takai. 2012. Microbial diversity in deep-sea methane seep sediments presented by SSU rRNA gene tag sequencing. Microbes Environ. 27:382-390.

6. Schloss, P.D., S.L. Westcott, T. Ryabin, et al. 2009. Introducing mothur: open-source, platform-independent, community-supported software for describing and comparing microbial communities. Appl. Environ. Microbiol. 75:7537-7541.

7. Tamura, K., G. Stecher, D. Peterson, A. Filipski, and S. Kumar. 2013. MEGA6: Molecular Evolutionary Genetics Analysis version 6.0. Mol. Biol. Evol. 30:2725-2729.

8. Toki, T., R. Higa, A. Ijiri, U. Tsunogai, and J. Ashi. 2014. Origin and transport of pore fluids in the Nankai accretionary prism inferred from chemical and isotopic compositions of pore water at cold seep sites off Kumano. Earth Planets Space 66:137.

9. Tomaru, H., U. Fehn, Z. Lu, R. Takeuchi, F. Inagaki, H. Imachi, R. Kotani, R. Matsumoto, and K. Aoike. 2009. Dating of dissolved iodine in pore waters from the gas hydrate occurrence offshore Shimokita Peninsula, Japan: $^{129}I$ results from the D/V

*Chikyu* shakedown cruise. Resour. Geol. 59:359-373.

10. Tsuchiya, M., and K. Takahashi. 2009. Scientific report for cruise CK09-03 Expedition 905. JAMSTEC, Yokosuka.
